# Draft Genome Sequence of *Pasteurella multocida* subsp. *multocida* Strain PMTB, Isolated from a Buffalo

**DOI:** 10.1128/genomeA.00872-13

**Published:** 2013-10-17

**Authors:** Huan Yong Yap, Kamal Ghazali, Wan Fahmi Wan Mohamad Nazarie, Mohd Noor Mat Isa, Zunita Zakaria, Abdul Rahman Omar

**Affiliations:** Institute of Bioscience, University Putra Malaysia, Serdang, Selangor, Malaysiaa; Codon Genomics, Jalan Bandar Lapan Belas, Pusat Bandar Puchong, Puchong, Selangor, Malaysiab; Malaysia Genome Institute, Jalan Bangi, Kajang, Selangor, Malaysiac; Faculty of Veterinary Medicine, University Putra Malaysia, Serdang, Selangor, Malaysiad

## Abstract

*Pasteurella multocida* serotypes B:2 and E:2 are the main causative agents of ruminant hemorrhagic septicemia in Asia and Africa, respectively. *Pasteurella multocida* strain PMTB was isolated from a buffalo with hemorrhagic septicemia and has been determined to be serotype B:2. Here we report the draft genome sequence of strain PMTB.

## GENOME ANNOUNCEMENT

Strains of *Pasteurella multocida* subsp. *multocida* are divided into five serogroups (A, B, D, E, and F) based on capsular type and into 16 serotypes (1 through 16) based on types of lipopolysaccharides (1). Each *P. multocida* serotype is generally associated with, but not restricted to, a specific host (2). The pathogenicity of *P. multocida* is associated with various virulence factors, which include capsules, lipopolysaccharides, adhesions, toxins, siderophores, and outer membrane proteins (3). However, the complete pathogenesis of hemorrhagic septicemia is still an enigma. Currently, four complete genome sequences of *P. multocida* subsp. *multocida* strains are available, those for Pm70 (GenBank accession number AE004439), 3480 (accession number CP001409), HN06 (accession number CP003313), and 36950 (accession number CP003022), which belong to serotypes F, A, D, and A, respectively.

The bacterium strain PMTB was obtained from the Veterinary Laboratory Service Unit (VLSU) of the Faculty of Veterinary Medicine, Universiti Putra Malaysia, where the bacteria were isolated from the carcass of a buffalo that died during an outbreak of hemorrhagic septicemia (HS) that occurred in 2003 in Kelantan (a state located in the northern part of peninsular Malaysia). Previous studies have shown that PMTB belongs to *P. multocida* serotype B:2 (4). Whole-genome sequencing of strain PMTB was performed by using an Illumina genome analyzer. A total of 7,760,284 single-end reads were generated from a genomic library with 300- to 500-bp fragments, providing approximately 100-fold genome coverage. The sequence was *de novo* assembled by use of the Velvet assembler, and 123 contigs were generated. The majority of the gaps between contigs were successfully closed by primer walking. The genome was annotated using the NCBI Prokaryotic Genomes Automatic Annotation Pipeline (PGAAP).

The improved draft genome sequence of strain PMTB has a genome size of 2,203,419 bp, a coding percentage of 87.3%, a G+C content of 40.44%, and 9 unclosed gaps. From the analysis of PGAAP results, strain PMTB contains 2,021 coding sequences (CDS) with an average size of 951 bp. Among the 2,021 CDS, 1,796 were assigned to functional clusters of orthologous groups (COGs), 51 had general function prediction only, and the remaining proteins had unknown functions.

The *toxA* gene, which is frequently detected in genomes of serotype D, was confirmed by PCR assay to be absent in the genome of strain PMTB. Furthermore, the integrative conjugative elements of *P. multocida* (ICE*Pmu*1) described by Michael and colleagues (5) were not found in the draft genome sequence of strain PMTB. A comparative analysis of the protein sequences encoded by the genome of the *P. multocida* serotype F:3 strain Pm70 and that of PMTB revealed that at 50% identity, 217 protein sequences encoded by strain PMTB represented unique proteins. Of these unique proteins, 22 were phage or transposon related.

### Nucleotide sequence accession numbers.

This whole-genome shotgun project has been deposited at DDBJ/EMBL/GenBank under the accession number AWTD00000000. The version described in this paper is version AWTD01000000.
